# Intravenous ferric carboxymaltose and ferric derisomaltose alter the intestinal microbiome in female iron-deficient anemic mice

**DOI:** 10.1042/BSR20231217

**Published:** 2023-09-25

**Authors:** Timo Rieg, Jianxiang Xue, Monica Stevens, Linto Thomas, James R. White, Jessica A. Dominguez Rieg

**Affiliations:** 1 Department of Molecular Pharmacology and Physiology, Hypertension and Kidney Research Center, University of South Florida, Tampa, FL 33612, U.S.A.; 2 James A. Haley Veterans’ Hospital, Tampa, FL 33612, U.S.A.; 3 Resphera Biosciences LLC, Baltimore, MD 21231, U.S.A.

**Keywords:** anemia, chronic kidney disease, inflammatory bowel disease, iron, microbiome

## Abstract

Iron deficiency anemia (IDA) is a leading global health concern affecting approximately 30% of the population. Treatment for IDA consists of replenishment of iron stores, either by oral or intravenous (IV) supplementation. There is a complex bidirectional interplay between the gut microbiota, the host’s iron status, and dietary iron availability. Dietary iron deficiency and supplementation can influence the gut microbiome; however, the effect of IV iron on the gut microbiome is unknown. We studied how commonly used IV iron preparations, ferric carboxymaltose (FCM) and ferric derisomaltose (FDI), affected the gut microbiome in female iron-deficient anemic mice. At the phylum level, vehicle-treated mice showed an expansion in *Verrucomicrobia*, mostly because of the increased abundance of *Akkermansia muciniphila*, along with contraction in *Firmicutes*, resulting in a lower *Firmicutes/Bacteroidetes* ratio (indicator of dysbiosis). Treatment with either FCM or FDI restored the microbiome such that *Firmicutes* and *Bacteroidetes* were the dominant phyla. Interestingly, the phyla *Proteobacteria* and several members of *Bacteroidetes* (e.g., *Alistipes*) were expanded in mice treated with FCM compared with those treated with FDI. In contrast, several *Clostridia* class members were expanded in mice treated with FDI compared with FCM (e.g., *Dorea* spp., *Eubacterium*). Our data demonstrate that IV iron increases gut microbiome diversity independently of the iron preparation used; however, differences exist between FCM and FDI treatments. In conclusion, replenishing iron stores with IV iron preparations in clinical conditions, such as inflammatory bowel disease or chronic kidney disease, could affect gut microbiome composition and consequently contribute to an altered disease outcome.

## Introduction

Iron is a vital component of many cellular processes (e.g., cell proliferation, respiration, energy production, oxygen transfer, and DNA synthesis). In adults, approximately 70% of the body’s iron stores (∼3–5 g) are utilized by erythrocytes, whereas most of the remaining iron is stored in the liver. Dietary iron absorption is limited to ∼1–2 mg/day; thus, the majority of iron needed (∼25–30 mg/day) must be recovered by reticuloendothelial macrophages that phagocytose senescent erythrocytes. Interestingly, both iron overload and deficiency can have detrimental effects on the body. Hemochromatosis, or excessive iron levels, can be toxic due to the formation of highly reactive hydroxyl radicals that can lead to organ injury. On the other hand, iron deficiency anemia (IDA) is one of the five leading causes of years lived with disability globally [1]. IDA is a significant public health concern for children and women; however, it is becoming increasingly recognized as a condition that worsens outcomes in patients with chronic disease and the elderly [2]. Treatment for IDA consists of iron store replenishment; oral iron is typically first line of treatment, followed by intravenous (IV) iron for more severe cases or when there is poor response or intolerable side effects from oral supplementation.

In the intestine, there is a complex interplay between the gut microbiota, host iron status, and dietary iron availability. Iron is essential for bacteria, which they acquire using one of three strategies: (1) secretion of siderophores, which are small, ferric iron-chelating compounds produced in response to low iron availability; (2) absorption of ferrous iron (Fe^+2^) after reducing ferric iron (Fe^+3^), if necessary; and (3) using host iron compounds such as heme and transferrin [3]. Not surprisingly, several studies have investigated the effect of iron-fortified and iron-deficient diets on the composition of the intestinal microbiota.

Iron availability has been shown to regulate virulence genes and promote proliferation of enteric pathogens [4]. In human studies, iron fortification was found to enhance the growth and virulence of enteric pathogens that led to diarrhea and intestinal inflammation in children [5,6]. Similarly, oral iron supplementation exacerbates colitis in patients with inflammatory bowel disease and in a murine dextran sodium sulfate (DSS)-induced colitis model, in addition to altering microbiota composition and diversity [7]. In contrast, the iron-sequestering protein lactoferrin, found in body fluids such as milk, tears, and saliva, has been shown to strengthen immunity against enteric pathogens by limiting their access to iron [8]. Further, numerous studies investigating the response to iron restriction have shown that the gut microbiome undergoes significant changes when iron is removed from the diet [9–13]. Many individual bacteria can rebound when iron levels are replenished to normal, but some iron-sensitive bacteria can be lost [14].

Importantly, host–microbe interactions are bidirectional, and the state of the gut microbiome can affect iron homeostasis in the host. Studies in mice treated with antibiotics and mice raised in germ-free conditions showed that the lack of a functional microbiome resulted in anemia [15]. This potentially related to the finding that enterocytes from germ-free mice exhibit decreased iron absorption and supports the hypothesis that under physiological conditions, the microbiome liberates a pool of iron from which the host benefits.

Although several studies have demonstrated the influence of dietary iron deficiency and oral iron supplementation on the gut microbiome, the effect of IV iron administration on the gut microbiome in IDA remains unknown. The aim of the current study was to determine how two commonly used IV iron therapy preparations, ferric carboxymaltose (FCM) and ferric derisomaltose (FDI), affect the gut microbiome of female iron-deficient anemic mice. Our data demonstrate that IV replenishment of iron drastically increases microbiome diversity, independent of the iron preparation used; however, some small differences exist between FCM and FDI treatments. This study has possible implications for how replenishing iron stores with IV iron preparations in other clinical conditions, such as inflammatory bowel disease or chronic kidney disease, could affect microbiome composition and consequently contribute to an altered disease outcome.

## Materials and methods

### Animals

All animal experiments were conducted at the University of South Florida in accordance with the Guide for the Care and Use of Laboratory Animals (National Institute of Health, Bethesda, MD, U.S.A.) and were approved by the Institutional Animal Care and Use Committee (R11592). Female 7-week-old C57Bl/6J mice were purchased from Jackson Laboratories (Bar Harbor, MA, U.S.A.). After acclimatization for 2 weeks, blood was collected from the retroorbital plexus under brief isoflurane inhalation anesthesia to determine the complete blood count (CBC; Vetscan, HM2 Hematology Analyzer, Abaxis, Union City, CA, U.S.A.). Mice were switched from a regular diet (TD.2018, Envigo, Madison, WI, U.S.A.) to an iron-deficient diet (TD.80396, Envigo) for 5 weeks, followed by IV bleeding (0.7% of body weight) for three consecutive days. On day 0 (the last day of bleeding), blood samples were analyzed for CBC. Mice were randomized to vehicle (saline, 2 µl/g body weight; *n*=8), FCM (20 mg/kg; *n*=9), or FDI (20 mg/kg; *n*=8) treatment groups via retro-orbital injection on days 0 and 7. Only the mice in the same treatment group were housed together. On day 14, all mice were killed via isoflurane overdose followed by confirmation of euthanasia via vital tissue harvest. Blood and feces were also collected. After centrifugation, plasma iron was analyzed using Iron Reagent (Pointe Scientific, Canton, MI, U.S.A.).

### Sample collection, and shotgun metagenomic sequencing and quality control of reads

Fecal pellets were collected in collection tubes containing DNA stabilization buffer. Whole metagenome shallow shotgun sequencing (at least 2 million paired-end reads per sample) was performed using the Illumina miSeq or Illumina NextSeq instrument (the instrument used is dependent on the number of samples in a batch, Illumina, San Diego, CA, U.S.A.). Samples were extracted using the Qiagen PowerMag Microbiome DNA Isolation kit (Hilden, Germany) on the King Fisher automated platform (Thermo Scientific, Waltham, MA, U.S.A.). Isolated DNA was quantitated using a fluorescent concentration assay and normalized to prepare for library preparation using the Illumina Nextera XT DNA Library prep recommendations. The runs were spiked with 1% PhiX. Standard processing used 2 × 150 base pair paired-end sequencing with dual 8 base pair indexes. The instrument run took approximately 29 h. Criteria for acceptable results: The final run must have a cluster density of 180–230 K/mm^2^ with >80% of clusters passing the filter, and at least 75% of bases must call at a minimum Phred score of Q30 (99.5%).

### Data analysis and statistics

The One Codex Database consists of approximately 114,000 complete microbial genomes, including 62,000 distinct bacterial genomes, 48,000 viral genomes, and approximately 4,000 fungal, archaeal, and eukaryotic genomes. The human genome was included to screen out host reads, and a complete list of references is available in the One Codex application at https://app.onecodex.com/references. The database was assembled from both public and private sources, with a combination of automated and manual curation steps to remove low-quality or mislabeled records. The comparison of a microbial sample against the One Codex Database consists of three sequential steps. First, every individual NGS read was compared against the One Codex Database by exact alignment using *k*-mers, where *k* = 31 ([16,17] for details on *k*-mer-based classification). The *k*-mer classification results were filtered based on the relative frequency of unique *k*-mers in the sample, and sequencing artifacts were filtered out of the samples. This filtering only removes probable sequencing or reference genome-based artifacts and does not filter out low-abundance or low-confidence hits. Finally, the relative abundance of each microbial species was estimated based on the depth and coverage of sequencing across all the available reference genomes. Microbial profiles were generated using the OneCodex analysis platform using the targeted loci module, with summarization at the phylum and species levels. The results were normalized to an even level of coverage using subsampling without replacement (11,000 observations per sample). Statistical analyses were performed using R Statistical Environment (v.3.5.3). Normalized species-level profiles were used to calculate α- and β-diversity measures using the vegan package in R. Principal coordinate analysis (PCoA) was performed using the ape R package. The PERMANOVA calculations were performed using the *adonis* function in vegan. Differential abundance analysis included calculation of the Mann–Whitney *U-*test and Welch’s *t*-test with log10 transformed values. Unsupervised clustering with a heatmap overlay applies the heatmap function in R with the Euclidean distance metric. Linear discriminant analysis (LDA) effect size (LEfSe) [18] was applied to the normalized taxonomic data to compare taxonomic membership between genotypes, followed by visualization of identified taxa by cladogram and individual LDA scores. Additional visualizations of stacked histograms and boxplots were performed using the ggplot2 package in R.

For blood parameters, data are expressed as mean ± SEM. One-way ANOVA followed by the Tukey multiple comparison test was used to test for significant differences between treatment groups. All data were analyzed via GraphPad Prism, v. 8.3 or SigmaPlot, v. 12.5. Significance was considered at *P*<0.05.

## Results

### Intravenous iron administration corrects iron deficiency in a mouse model of IDA

**Figure 1:**
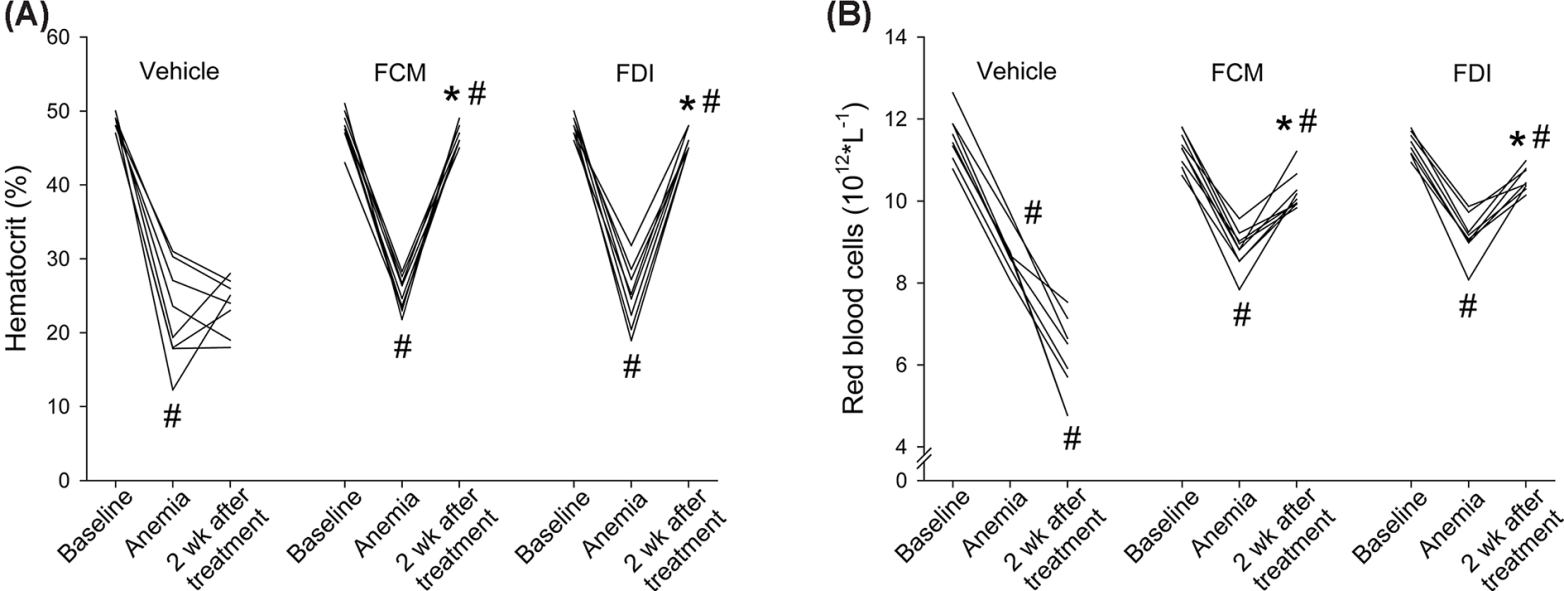
FCM and FDI administration correct iron deficiency anemia At baseline, prior to switching from normal chow to a low iron diet, no differences were observed in hematocrit (**A**) or red blood cell count (RBC; **B**) between the vehicle, FCM, and FDI treatment groups. Bleeding-induced anemia, as seen by reduced hematocrit (A) and RBC (B), to similar levels in all groups. After 14 days, mice treated with FCM and FDI showed increases in their hematocrit (A) and RBC (B), whereas vehicle-treated mice remained anemic with low hematocrit (A) and further decreased RBC (B). Data are expressed as mean±SEM and were analyzed by repeated measures two-way ANOVA followed by Tukey’s multiple comparison test. *P<0.05, vs. vehicle, same condition; ^#^*P*<0.05, vs. previous condition in the same treatment group. *N* = 8–9/group.

At baseline, prior to switching mice from normal chow to a low iron diet, no differences were observed in hematocrit (48 ± 0.4 vs. 48 ± 0.6 vs. 48 ± 0.4%, *NS*) or red blood cell count (RBC, 11 ± 0.2 vs. 11 ± 0.1 vs. 11 ± 0.1 10^12^*L^−1^, *NS*) between vehicle, FCM and FDI treatment groups, respectively (Figure 1A,B). Bleeding reduced hematocrit (22 ± 2 vs. 25 ± 1 vs. 25 ± 1%; *P*<0.05 vs. baseline) and RBC (8.8 ± 0.2 vs. 8.8 ± 0.2 vs. 9.1 ± 0.2 10^12^*L^−1^, *P<*0.05 vs. pre-bleeding) to similar levels in all groups, consistent with the induction of microcytic hypochromic anemia (Figure 1A,B). After 14 days, mice treated with FCM and FDI showed significant increases in their hematocrit (47 ± 0.4 and 46 ± 0.3%, respectively; *P*<0.05 vs. anemia) and RBC (10 ± 0.1 and 11 ± 0.1 10^12^*L^−1^, respectively; *P*<0.05 vs. anemia) (Figure 1A,B). In contrast, in vehicle-treated mice, hematocrit was not significantly different (24 ± 1%; *NS* vs. anemia), and RBC decreased significantly further (6.1 ± 0.3 10^12^*L^−1^, *P*<0.05 vs. anemia) (Figure 1A,B). Compared to vehicle-treated mice, plasma iron levels were ∼10-fold higher in mice treated with either FCM or FDI (1.1 ± 0.5 μmol/L vs. 13.6 ± 1.5 μmol/L vs. 10.0 ± 0.7 μmol/L, respectively; *P<*0.05 vs. vehicle), but there was no significant difference between FCM and FDI treatments. These experiments established the successful correction of IDA in response to FCM and FDI treatment compared with the vehicle.

### Intravenous iron administration distinctly impacts microbiome diversity

**Figure 2:**
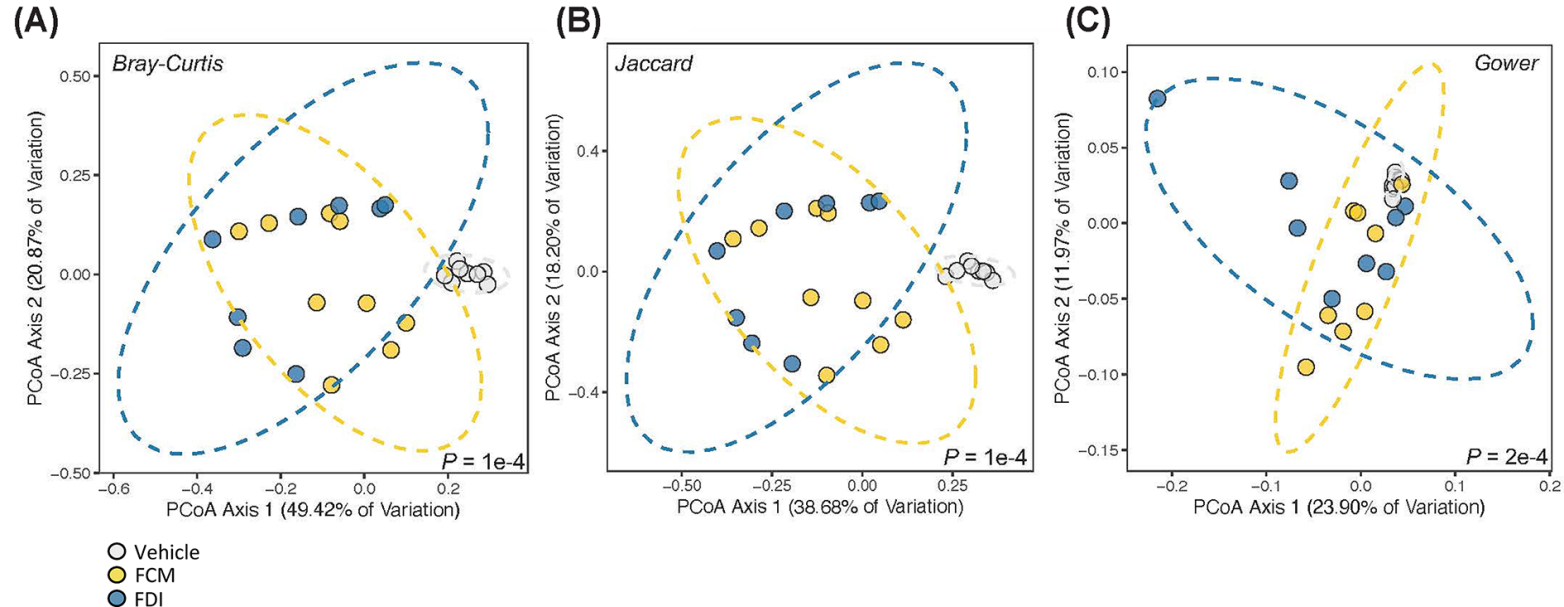
β-Diversity principal coordinates analysis (PCoA) in vehicle-, FCM-, and FDI-treated mice Microbiota composition was significantly different among the three treatment groups (*n* = 8–9/group) according to three different measures of beta diversity: (**A**) Bray–Curtis, (**B**) Jaccard, and (**C**) Gower distances (PERMANOVA *P*-value displayed per panel). The percentage of variation explained per PCoA axis is displayed with the title axis.

**Figure 3:**
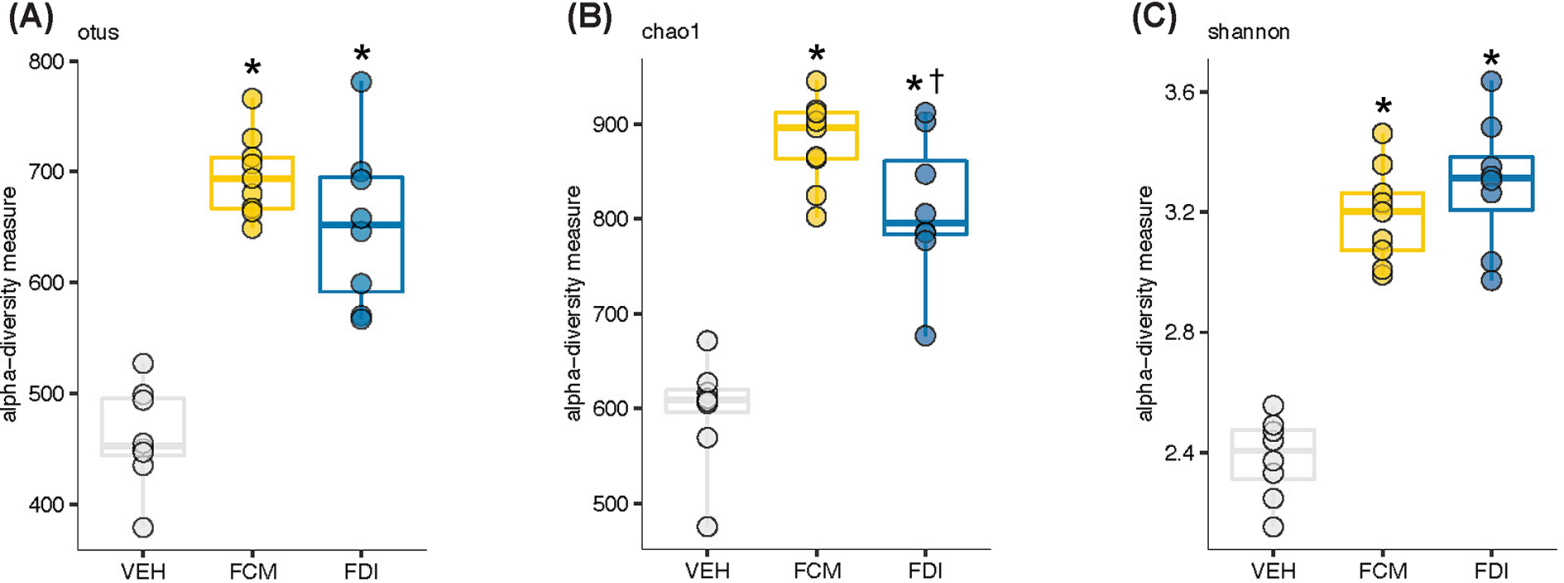
Differential α-diversity levels in vehicle-, FCM- and FDI-treated mice α-Diversity analysis suggests significant differences in otus (**A**) and chao1 (**B**) richness estimators and Shannon diversity (**C**) between vehicle-treated mice (*n*=8) and FCM- (*n*=9) or FDI-treated (*n*=8) mice (Mann–Whitney *U-*test). *P<0.05 vs. vehicle; ^†^*P*<0.05 vs. FCM.

Analysis of gut microbiome β-diversity, a measure of diversity differences between treatments, revealed that FCM- and FDI-treated mice had distinct gut microbiome signatures that clustered differently when compared with their age- and sex-matched vehicle treatment groups. Pairwise compositional dissimilarity analysis using Bray–Curtis (*P*=0.0001), Jaccard (*P*=0.0001), and Gower (*P*=0.0002) indices showed significant differences between the vehicle and treatment groups (Figure 2). Further analysis of microbial α-diversity, a measure of variance within a specific group, showed that the FCM and FDI treatment groups harbored distinct populations of gut microbes compared with the vehicle treatment group. Operational taxonomic units (OTU richness, taxa level, FCM vs. vehicle: *P*<0.001; FDI vs. vehicle: *P*<0.001), Chao1 (richness estimator, phylum level, FCM vs. vehicle: *P*<0.001; FDI vs. vehicle: *P*<0.001), and Shannon index (reflects species numbers and evenness of species abundance, FCM vs. vehicle: *P*<0.001; FDI vs. vehicle: *P*<0.001) all showed a significantly greater microbial diversity in FCM- and FDI-treated mice than in vehicle-treated mice (Figure 3). α-Diversity was significantly lower in the FDI versus FCM treatment at the phylum level (Chao1: *P*=0.046); however, no significant differences were observed at the taxa level or in the evenness of species abundance.

### Differential abundance of microbiota in response to IV iron treatment

**Figure 4:**
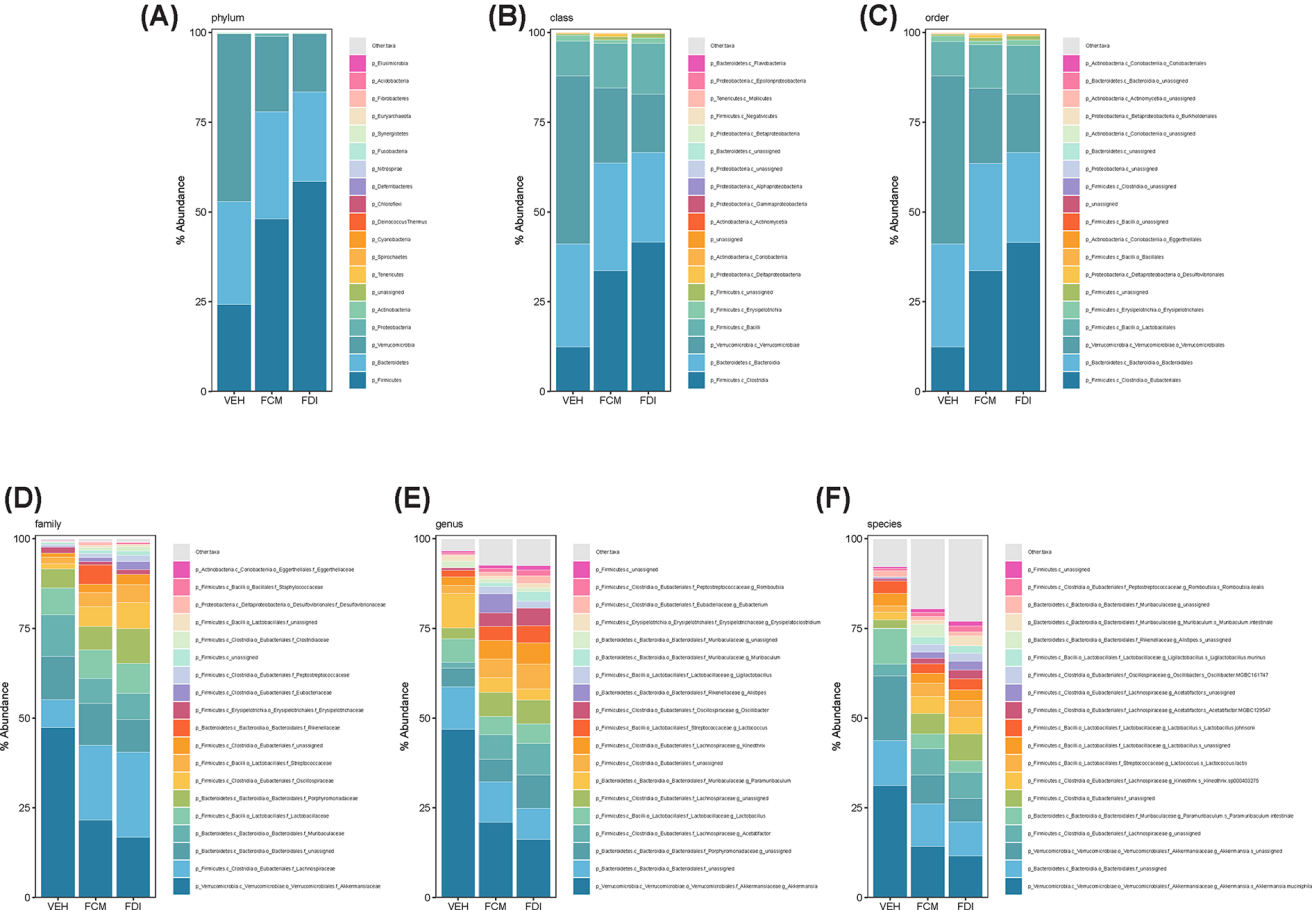
Taxonomic composition distribution histograms of gut microbiota from vehicle-, FCM-, and FDI-treated mice Composition of gut microbiota at the phylum (**A**), class (**B**), order (**C**), family (**D**), genus (**E**), and species (**F**) levels in vehicle- (*n*=8), FCM- (*n*=9), and FDI-treated (*n*=8) mice.

We systematically analyzed the microbial composition distribution and differential abundance from phylum to species levels between vehicle-, FCM-, and FDI-treated mice. Compared with the vehicle treatment, FCM- and FDI-treated mice showed significant differential distributions across taxonomic levels (Figure 4 and Table 1). At the phylum level (Figure 4A), *Verrucomicrobia* (47 ± 3%), *Bacteroidetes* (29 ± 1%), and *Firmicutes* (24 ± 2%) were the three most abundant phyla in the vehicle-treated mice. In contrast, correction of IDA with either FCM or FDI resulted in a significant increase in *Firmicutes* compared with vehicle (48 ± 5% and 59 ± 4%, respectively; *P*<0.001 vs. vehicle). However, there was no significant difference in *Firmicutes* abundance between the FCM- and FDI-treated mice. There was also a significantly lower abundance of *Verrucomicrobia* in mice treated with either FCM or FDI compared with vehicle (21 ± 3% and 16 ± 4%, respectively; *P*<0.01 vs. vehicle) and no difference between the IV iron treatment groups. Interestingly, the abundance of *Bacteroidetes* was not different between vehicle-, FCM-, and FDI-treated mice (29 ± 1% vs. 30 ± 3% vs. 25 ± 2%, respectively; *NS*). A decreased *Firmicutes/Bacteroidetes* (F/B) ratio is commonly accepted as an indicator of dysbiosis. We found that the F/B ratio in vehicle-treated mice was significantly lower compared with FCM or FDI treatments (0.9 ± 0.1 vs 1.9 ± 0.3 vs 2.4 ± 0.3; *P*<0.05 vs. vehicle). The phylum *Proteobacteria* was contracted in vehicle-treated mice compared with FCM- and FDI-treated mice (0.01 ± 0.0% vs. 0.80 ± 0.4% vs. 0.04 ± 0.01%, respectively; *P*<0.01 vs. vehicle), and there was a significant expansion of *Proteobacteria* in FCM-treated mice compared with FDI-treated mice (0.80 ± 0.4% vs. 0.04 ± 0.01%, *P*<0.05). There was no significant difference in the abundance of the phylum *Actinobacteria* between vehicle-, FCM-, or FDI-treated mice (0.23 ± 0.02% vs. 0.18 ± 0.02% vs. 0.19 ± 0.03%, *NS*).

**Table 1:** Differential abundance of microbiota across taxonomic levels between vehicle-, FCM-, and FDI-treated mice

Taxa	Taxonomic description	Relative abundance (% ± SEM)			*P-*value	*P-*value	*P-*value	FCM change vs. vehicle	FDI change vs. vehicle	FCM change vs. FDI
		Vehicle (*n*=8)	FCM (*n*=9)	FDI (*n*=8)	FCM vs. Vehicle	FDI vs. Vehicle	FCM vs. FDI			
Phylum	*Actinobacteria*	0.23 ± 0.02	0.18 ± 0.02	0.19 ± 0.03	0.08056	0.13837	0.85869	-	-	-
	*Bacteroidetes*	28.66 ± 1.38	29.87 ± 3.36	24.97 ± 1.82	0.98593	0.11049	0.29640	-	-	-
	*Firmicutes*	24.26 ± 1.76	48.07 ± 4.47	58.46 ± 4.00	0.00010	0.00000	0.10473	↑	↑	-
	*Firmicutes/Bacteroidetes*	0.86 ± 0.07	1.90 ± 0.33	2.44 ± 0.26	0.03390	0.00150	0.42060	↑	↑	-
	*Proteobacteria*	0.01 ± 0.00	0.82 ± 0.39	0.04 ± 0.01	0.00230	0.00082	0.03197	↑	↑	↑
	*Verrucomicrobia*	46.8 ± 2.50	20.97 ± 2.73	16.24 ± 3.94	0.00072	0.00330	0.23150	↓	↓	-
Class	*Coriobacteriia*	0.23 ± 0.02	0.17 ± 0.02	0.17 ± 0.03	0.05617	0.08959	0.73609	-	-	-
	*Bacteroidia*	28.66 ± 1.38	29.86 ± 3.37	24.95 ± 1.82	0.98330	0.10915	0.29561	-	-	-
	*Bacilli*	9.73 ± 1.67	12.45 ± 2.54	14.20 ± 1.97	0.37336	0.19490	0.59685	-	-	-
	*Clostridia*	12.45 ± 1.16	33.70 ± 4.38	41.61 ± 5.41	0.00009	0.00002	0.30036	↑	↑	-
	*Erysipelotrichia*	1.57 ± 0.11	1.01 ± 0.17	1.48 ± 0.29	0.01623	0.42269	0.15630	↓	-	-
	*Deltaproteobacteria*	0.00 ± 0.00	0.8 ± 0.39	0.01 ± 0.00	0.00489	0.06405	0.01769	↑	-	↑
	*Verrucomicrobiae*	46.80 ± 2.50	20.96 ± 2.73	16.24 ± 3.94	0.00071	0.00331	0.23186	↓	↓	-
Order	*Eggerthellales*	0.23 ± 0.02	0.17 ± 0.02	0.17 ± 0.03	0.05069	0.07210	0.66686	-	-	-
	*Bacteroidales*	28.65 ± 1.38	29.87 ± 3.37	24.97 ± 1.82	0.98861	0.11162	0.29651	-	-	-
	*Bacillales*	0.04 ± 0.02	0.21 ± 0 .05	0.47 ± 0.16	0.00016	0.00012	0.21012	↑	↑	-
	*Lactobacillales*	9.62 ± 1.66	12.16 ± 2.52	13.62 ±1.99	0.41006	0.27049	0.68228	-	-	-
	*Eubacteriales*	12.43 ± 1.15	33.66 ± 4.38	41.58 ± 5.40	0.00009	0.00002	0.29800	↑	↑	-
	*Erysipelotrichales*	1.57 ± 0.11	1.01 ± 0.17	1.49 ± 0.29	0.01666	0.43554	0.15394	↓	-	-
	*Desulfovibrionales*	0.00 ± 0.00	0.79 ± 0.39	0.01 ± 0.00	0.00680	0.24838	0.01521	↑	-	↑
	*Verrucomicrobiales*	46.81 ± 2.50	20.96 ± 2.73	16.23 ± 3.94	0.00071	0.00329	0.23185	↓	↓	-
Family	*Eggerthellaceae*	0.23 ± 0.02	0.17 ± 0.02	0.17 ±0.03	0.06955	0.09495	0.71684	-	-	-
	*Bacteroidaceae*	0.02 ± 0.00	0.20 ± 0.05	0.09 ± 0.04	0.00002	0.29274	0.02521	↑	-	↑
	*Muribaculaceae*	11.65 ± 0.54	6.91 ± 0.99	7.23 ± 1.17	0.00472	0.00616	0.77695	↓	↓	-
	*Porphyromonadaceae*	5.23 ± 0.37	6.51 ± 1.85	9.70 ± 1.07	0.34033	0.00256	0.06623	-	↑	-
	*Rikenellaceae*	0.02 ± 0.02	5.46 ± 2.25	0.01 ± 0.00	0.03047	0.64600	0.02213	↑	-	↑
	*Staphylococcaceae*	0.03 ± 0.01	0.20 ± 0.05	0.48 ± 0.17	0.00023	0.00013	0.19824	↑	↑	-
	*Lactobacillaceae*	7.54 ± 1.50	7.99 ± 2.21	8.40 ± 1.43	0.82126	0.94153	0.93208	-	-	-
	*Streptococcaceae*	1.84 ± 0.18	4.02 ± 0.53	4.99 ± 0.60	0.00070	0.00008	0.26042	↑	↑	-
	*Clostridiaceae*	0.12 ± 0.02	0.78 ± 0.10	1.30 ± 0.29	0.00010	0.00002	0.15745	↑	↑	-
	*Eubacteriaceae*	0.31 ± 0.07	1.10 ± 0.28	2.29 ± 0.39	0.00190	0.00002	0.01883	↑	↑	↓
	*Lachnospiraceae*	7.73 ± 1.10	20.85 ± 3.35	23.79 ± 3.28	0.00097	0.00011	0.46743	↑	↑	-
	*Oscillospiraceae*	1.51 ± 0.31	5.48 ± 0.83	7.27 ± 1.28	0.00026	0.00016	0.40026	↑	↑	-
	*Peptostreptococcaceae*	0.57 ± 0.07	1.12 ± 0.16	1.67 ± 0.19	0.00450	0.00008	0.06692	↑	↑	-
	*Erysipelotrichaceae*	1.57 ± 0.11	0.90 ± 0.16	1.33 ± 0.29	0.00702	0.18474	0.16473	↓	-	-
	*Turicibacteraceae*	0.02 ± 0.01	0.13 ± 0.02	0.21 ± 0.05	0.00148	0.00068	0.16356	↑	↑	-
	*Desulfovibrionaceae*	0.00 ± 0.00	0.83 ± 0.41	0.01 ± 0.00	0.00608	0.16840	0.01565	↑	-	↑
	*Akkermansiaceae*	47.37 ± 2.55	21.62 ± 2.81	16.77 ±4.01	0.00084	0.00342	0.23730	↓	↓	-
Genus	*Adlercreutzia*	0.22 ± 0.02	0.17 ± 0.02	0.17 ± 0.03	0.05021	0.07475	0.75731	-	-	-
	*Phocaeicola*	0.01 ± 0.00	0.15 ± 0.04	0.04 ± 0.02	0.00059	0.27793	0.01629	↑	-	↑
	*Duncaniella*	0.23 ± 0.01	0.62 ± 0.07	0.19 ± 0.02	0.00018	0.04907	0.00003	↑	↓	↑
	*Muribaculum*	0.06 ± 0.00	1.07 ± 0.37	2.63 ± 0.74	0.01230	0.01710	0.50112	↑	↑	-
	*Paramuribaculum*	9.55 ± 0.46	4.06 ± 1.22	3.09 ± 1.53	0.01878	0.00928	0.13847	↓	↓	-
	*Alistipes*	0.02 ± 0.02	5.28 ± 2.19	0.01 ± 0.00	0.03129	0.52135	0.02021	↑	-	↑
	*Staphylococcus*	0.01 ± 0.00	0.06 ± 0.01	0.12 ± 0.03	0.00005	0.00001	0.08950	↑	↑	-
	*Enterococcus*	0.10 ± 0.02	0.01 ± 0.00	0.13 ± 0.11	0.00059	0.12914	0.13524	↓	-	-
	*Lactobacillus*	6.61 ± 1.34	4.98 ± 2.05	5.39 ± 1.51	0.10028	0.23449	0.91638	-	-	-
	*Ligilactobacillus*	0.01 ± 0.01	2.03 ± 0.72	1.91 ± 1.00	0.00002	0.10090	0.09477	↑	-	-
	*Lactococcus*	1.82 ± 0.18	3.89 ± 0.52	4.79 ± 0.60	0.00106	0.00017	0.29937	↑	↑	-
	*Clostridium*	0.05 ± 0.01	0.38 ± 0.05	0.73 ± 0.24	0.00021	0.00003	0.13682	↑	↑	-
	*Hungatella*	0.07 ± 0.02	0.37 ± 0.06	0.49 ± 0.07	0.00059	0.00026	0.44106	↑	↑	-
	*Eubacterium*	0.30 ± 0.07	1.07 ± 0.27	2.18 ± 0.36	0.00225	0.00003	0.01876	↑	↑	↓
	*Emergencia*	0.09 ± 0.06	0.06 ± 0.02	0.12 ± 0.03	0.81054	0.08750	0.04594	-	-	↓
	*Acetatifactor*	1.62 ± 0.30	6.85 ± 1.33	8.76 ± 1.05	0.00097	0.00001	0.20045	↑	↑	-
	*Dorea*	0.45 ± 0.08	0.93 ± 0.12	0.74 ± 0.10	0.00253	0.03642	0.24906	↑	↑	-
	*Kineothrix*	2.37 ± 0.36	5.12 ± 0.83	5.92 ± 1.11	0.00498	0.00582	0.68999	↑	↑	-
	*Schaedlerella*	0.12 ± 0.01	0.31 ± 0.05	0.43 ± 0.06	0.00174	0.00007	0.19766	↑	↑	-
	*Acutalibacter*	0.09 ± 0.01	0.35 ± 0.04	0.47 ± 0.14	0.00002	0.00028	0.67343	↑	↑	-
	*Anaerotruncus*	0.08 ± 0.02	0.28 ± 0.06	0.56 ± 0.15	0.00280	0.00699	0.00600	↑	↑	↓
	*Angelakisella*	0.14 ± 0.03	0.41 ± 0.05	0.43 ± 0.07	0.00044	0.00339	0.83197	↑	↑	-
	*Oscillibacter*	0.80 ± 0.18	3.87 ± 0.70	4.99 ± 0.80	0.00091	0.00034	0.36353	↑	↑	-
	*Romboutsia*	0.57 ± 0.07	1.09 ± 0.16	1.60 ± 0.19	0.00604	0.00015	0.08252	↑	↑	-
	*Erysipelatoclostridium*	1.54 ± 0.11	0.86 ± 0.16	1.26 ± 0.29	0.00601	0.08298	0.14406	↓	-	-
	*Turicibacter*	0.02 ± 0.01	0.13 ± 0.02	0.20 ± 0.05	0.00150	0.00070	0.18790	↑	↑	-
	*Desulfovibrio*	0.00 ± 0.00	0.77 ± 0.38	0.00 ± 0.00	0.01531	0.54529	0.02101	↑	-	↑
	*Akkermansia*	46.82 ± 2.50	20.96 ± 2.72	16.24 ± 3.94	0.00071	0.00329	0.23175	↓	↓	-
Species	*Phocaeicola vulgatus*	0.01 ± 0.00	0.15 ± 0.04	0.04 ± 0.02	0.00284	0.67880	0.02156	↑	-	↑
	*Duncaniella sp001689425*	0.00 ± 0.00	0.4 ±0 .06	0.00 ± 0.00	0.00005	N/A	0.00005	↑	-	↑
	*Paramuribaculum intestinale*	10.00 ± 0.50	4.09 ± 1.23	3.22 ± 1.61	0.01725	0.00987	0.04293	↓	↓	↑
	*Alistipes sp002362235*	0.00 ± 0.00	1.39 ± 0.55	0.00 ± 0.00	0.07340	0.35062	0.03535	-	-	↑
	*Lactobacillus johnsonii*	3.42 ± 0.70	2.67 ± 1.09	3.00 ± 0.85	0.12314	0.29626	0.85684	-	-	-
	*Ligilactobacillus murinus*	0.01 ± 0.01	2.02 ± 0.70	1.98 ± 1.04	0.00006	0.08765	0.11215	↑	-	-
	*Lactococcus cremoris*	0.05 ± 0.01	0.13 ± 0.02	0.17 ± 0.02	0.00026	0.00004	0.29612	↑	↑	-
	*Lactococcus lactis*	1.69 ± 0.17	3.69 ± 0.55	4.66 ± 0.62	0.00168	0.00016	0.26337	↑	↑	-
	*Clostridium MGBC131118*	0.03 ± 0.01	0.17 ± 0.03	0.28 ± 0.07	0.01469	0.00785	0.16531	↑	↑	-
	*Clostridium MGBC164501*	0.00 ± 0.00	0.01 ± 0.01	0.23 ± 0.15	0.02302	0.01882	0.33329	↑	↑	-
	*Clostridium sp.MD294*	0.00 ± 0.00	0.14 ± 0.03	0.15 ± 0.03	0.00005	0.00004	0.79991	↑	↑	-
	*Hungatella sp002358555*	0.03 ± 0.01	0.15 ± 0.03	0.22 ± 0.04	0.01551	0.00935	0.25418	↑	↑	-
	*Eubacterium MGBC000141*	0.02 ± 0.01	0.14 ± 0.03	0.22 ± 0.04	0.00203	0.09248	0.26266	↑	-	-
	*Eubacterium MGBC101131*	0.19 ± 0.08	0.67 ± 0.29	1.83 ± 0.31	0.40866	0.00323	0.01686	-	↑	↓
	*Eubacterium MGBC164771*	0.05 ± 0.01	0.13 ± 0.02	0.18 ± 0.02	0.00043	0.00000	0.06631	↑	↑	-
	*Emergencia MGBC000042*	0.09 ± 0.06	0.06 ± 0.02	0.13 ± 0.03	0.81145	0.06985	0.03797	-	-	↓
	*Acetatifactor MGBC113998*	0.01 ± 0.00	0.15 ± 0.05	0.14 ± 0.04	0.44645	0.11205	0.51944	-	-	-
	*Acetatifactor MGBC118768*	0.02 ± 0.00	0.11 ± 0.02	0.17 ± 0.07	0.00000	0.37719	0.58867	↑	-	-
	*Acetatifactor MGBC129547*	0.58 ± 0.14	1.61 ± 0.44	2.49 ± 0.29	0.04237	0.00017	0.05287	↑	↑	-
	*Acetatifactor MGBC130773*	0.03 ± 0.02	1.23 ± 0.49	0.74 ± 0.32	0.00022	0.03666	0.19620	↑	↑	-
	*Acetatifactor MGBC130908*	0.11 ± 0.03	0.36 ± 0.09	0.98 ± 0.31	0.08436	0.01794	0.05576	-	↑	-
	*Acetatifactor MGBC146413*	0.14 ± 0.04	0.11 ± 0.03	0.42 ± 0.10	0.60758	0.48092	0.25857	-	-	-
	*Acetatifactor MGBC159247*	0.09 ± 0.02	0.44 ± 0.10	0.26 ± 0.04	0.00343	0.00254	0.47966	↑	↑	-
	*Acetatifactor MGBC162151*	0.01 ± 0.00	0.02 ± 0.01	0.51 ± 0.33	0.41893	0.24089	0.55542	-	-	-
	*Acetatifactor MGBC165149*	0.09 ± 0.08	0.38 ± 0.16	0.13 ± 0.07	0.02997	0.13758	0.45132	↑	-	-
	*Acetatifactor sp003612485*	0.11 ± 0.07	1.13 ± 0.24	1.14 ± 0.34	0.00282	0.24986	0.24597	↑	-	-
	*Dorea MGBC000089*	0.01± 0.01	0.08 ± 0.02	0.16 ± 0.03	0.02624	0.00001	0.03181	↑	↑	↓
	*Dorea MGBC000111*	0.00 ± 0.00	0.25 ± 0.08	0.01 ± 0.01	0.00090	0.96606	0.00175	↑	-	↑
	*Dorea MGBC107888*	0.01 ± 0.00	0.28 ± 0.05	0.12 ± 0.03	0.00000	0.00009	0.01558	↑	↑	↑
	*Dorea MGBC109699*	0.35 ± 0.07	0.06 ± 0.01	0.15 ± 0.02	0.00002	0.00579	0.00006	↓	↓	↓
	*Kineothrix MGBC130615*	0.12 ± 0.05	0.10 ± 0.01	0.10 ± 0.02	0.67121	0.87530	0.71702	-	-	-
	*Kineothrix MGBC162921*	0.06 ± 0.01	0.39 ± 0.12	1.45± 0.88	0.03176	0.00870	0.08714	↑	↑	-
	*Kineothrix sp000403275*	2.20 ± 0.32	4.85 ± 0.88	4.63 ± 0.74	0.00716	0.00876	0.93568	↑	↑	-
	*Schaedlerella MGBC000001*	0.03 ± 0.01	0.13 ± 0.03	0.16 ± 0.03	0.00744	0.00038	0.25232	↑	↑	-
	*Acutalibacter MGBC129708*	0.01 ± 0.00	0.12 ± 0.02	0.10 ± 0.06	0.00001	0.38348	0.04586	↑	-	↑
	*Acutalibacter muris*	0.05 ± 0.01	0.19 ± 0.03	0.32 ± 0.08	0.00070	0.00018	0.19371	↑	↑	-
	*Anaerotruncus sp000403395*	0.05 ± 0.01	0.15 ± 0.03	0.29 ± 0.08	0.00269	0.42099	0.84444	↑	↑	-
	*Angelakisella MGBC131977*	0.03 ± 0.01	0.09 ± 0.01	0.17 ± 0.04	0.00095	0.00128	0.24375	↑	↑	-
	*Angelakisella MGBC136623*	0.11 ± 0.03	0.35 ± 0.05	0.28 ± 0.05	0.00207	0.00772	0.45755	↑	↑	-
	*Oscillibacter MGBC114113*	0.19 ± 0.09	0.61 ± 0.19	0.71 ± 0.22	0.01793	0.10053	0.41705	↑	↑	-
	*Oscillibacter MGBC104191*	0.07 ± 0.03	0.18 ± 0.03	0.25 ± 0.07	0.00397	0.00350	0.63321	↑	↑	-
	*Oscillibacter MGBC129725*	0.01 ± 0.01	0.24 ± 0.16	0.09 ± 0.08	0.82775	0.36337	0.36977	-	-	-
	*Oscillibacter MGBC161747*	0.02 ± 0.01	2.14 ± 0.56	2.28 ± 0.49	0.00033	0.00029	0.67504	↑	↑	-
	*Oscillibacter MGBC163303*	0.01 ± 0.00	0.31 ± 0.11	0.42 ± 0.29	0.00043	0.08132	0.29944	↑	↑	-
	*Oscillibacter sp000403435*	0.15 ± 0.06	0.07 ± 0.02	0.38 ± 0.14	0.79230	0.09172	0.02806	-	-	↓
	*Romboutsia ilealis*	0.57 ± 0.07	1.14 ± 0.19	1.71 ± 0.21	0.00935	0.00014	0.07895	↑	↑	-
	*Erysipelatoclostridium cocleatum*	1.12 ± 0.08	0.65 ± 0.13	0.96 ± 0.22	0.00957	0.21778	0.18450	↓	-	-
	*Desulfovibrio MGBC000161*	0.00 ± 0.00	0.77 ± 0.38	00.00 ± 0.00	0.03537	0.67190	0.04396	↑	-	↑
	*Akkermansia muciniphila*	31.20 ± 1.60	14.26 ± 1.84	11.60 ± 2. 80	0.00077	0.00436	0.28006	↓	↓	-

Note: The symbol ‘↑’ indicates expansion; ‘↓’ indicates contraction in FCM- and FDI-treated mice compared with vehicle-treated mice; ‘-’ indicates no difference between treatment groups. Bacteria with at least a 0.01% abundance in the treatment group are shown. Unadjusted *t*-test *P*-values are shown.

At the class level (Figure 4B and Table 1), the expansion of *Verrucomicrobiae* (47 ± 3% vs. 21 ± 3% vs. 16 ± 4%; *P*<0.01 vs. vehicle) and the contraction of *Clostridia* (13 ± 1% vs. 34 ± 4% vs. 42 ± 5%; *P*<0.001 vs. vehicle) were primarily responsible for the differences between vehicle and FCM or FDI treatments, respectively. Compared with the vehicle, *Erysipelotrichia* was contracted in the FCM-treated mice (2 ± 0.1% vs. 1 ± 0.2%; *P*<0.05), but there was no difference between vehicle- and FDI-treated mice (2 ± 0.1% vs. 2 ± 0.3%; *NS*). *Deltaproteobacteria* was undetectable in vehicle-treated mice but was significantly expanded in FCM-treated mice compared with FDI-treated mice (1 ± 0.4% vs. 0.01 ± 0.0%, *P*<0.05). The abundance of the classes *Bacteroidia* (29 ± 1% vs. 30 ± 3% vs. 25 ± 2%; *NS*), *Bacilli* (10 ± 2% vs. 13 ± 3% vs. 14 ± 2%; *NS*), and *Coriobacteriia* (0.2 ± 0.02% vs. 0.2 ± 0.02% vs. 0.2 ± 0.03%; *NS*) were not significantly different between the vehicle, FCM, and FDI treatment groups, respectively.

The most abundant order (Figure 4C and Table 1) in vehicle-treated mice was *Verrucomicrobiales* (47 ± 3%), which showed an expansion compared to FCM and FDI treatments (21 ± 3% and 16 ± 4%, respectively; *P*<0.01 vs. vehicle). In contrast, vehicle-treated mice showed significant contractions in *Eubacteriales* (12 ± 1%) and *Bacillales* (0.04 ± 0.01%) compared with mice treated with FCM (34 ± 4% and 0.2 ± 0.1%, respectively; *P*<0.001 vs. vehicle) and FDI (42 ± 5% and 0.5 ± 0.2%, respectively; *P*<0.001 vs. vehicle). Compared with vehicle, the order *Erysipelotrichales* was contracted only in FCM-treated mice (1.6 ± 0.1% vs. 1.0 ± 0.2%; *P*<0.05), but there was no difference between vehicle- and FDI-treated mice (1.6 ± 0.1% vs. 1.5 ± 0.3%; *NS*). *Desulfovibrionales* was undetectable in vehicle-treated mice but was significantly expanded in FCM-treated mice compared with FDI-treated mice (0.8 ± 0.4% vs. 0.01 ± 0.0%; *P*<0.05). *Bacteroidales* (29 ± 1% vs. 30 ± 3% vs. 25 ± 2%; *NS*), *Lactobacillales* (10 ± 2% vs. 12 ± 3% vs. 14 ± 2%; *NS*), and *Eggerthellales* (0.2 ± 0.02% vs. 0.2±0.02% vs. 0.2 ± 0.03%; *NS*) showed no significant differences between vehicle-, FCM-, or FDI-treated mice, respectively.

At the family level (Figure 4D and Table 1), significant expansions in the abundance of *Akkermansiaceae* (47 ± 3%) and *Muribaculaceae* (12 ± 1%) were observed in vehicle-treated mice compared with FCM- and FDI-treated mice (22 ± 3% and 17 ± 4%; 7 ± 1% and 7 ± 1%, respectively; *P*<0.01 vs vehicle). Significant contractions in 8 family members of the phyla *Firmicutes*, including but not limited to *Streptococcaceae* (1.8 ± 0.2%), *Lachnospiraceae* (8 ± 1%), and *Oscillospiraceae* (1.5 ± 0.3%), were observed in vehicle-treated mice compared with FCM- and FDI-treated mice (4 ± 1% and 5 ± 1%; 21 ± 3% and 24 ± 3%; 6 ± 1% and 7 ± 1%, respectively; *P*<0.001 vs. vehicle). Of note, significant expansions of *Bacteroidaceae* (0.2 ± 0.1%), *Rikenellaceae* (6 ± 2%), and *Desulfovibrionaceae* (1 ± 0.4%) were only observed in FCM-treated mice but not vehicle- or FDI-treated mice (0.02 ± 0.0% and 0.1 ± 0.04%; 0.02 ± 0.02% and 0.01 ± 0.0%; 0.0 ± 0.0% and 0.01 ± 0.0%, respectively; *P*<0.05 vs. FCM). In contrast, compared with vehicle-treated mice, *Erysipelotrichaceae* was contracted in FCM-treated mice (2 ± 0.1% vs. 1 ± 0.2%; *P*<0.01), while there were significant expansions in *Porphyromonadaceae* (5 ± 0.4% vs. 10 ± 1%; *P*<0.01) and *Eubacteriaceae* (0.3 ± 0.1% vs. 2 ± 0.4%; *P*<0.0001) in FDI-treated mice.

At the genus level (Figure 4E and Table 1), significant expansions in the abundance of *Akkermansia* (47 ± 3%, *P*<0.01) and *Paramuribaculum* (10 ± 1%; *P*<0.05) were observed in vehicle-treated mice compared with FCM- and FDI-treated mice (21 ± 3% and 16 ± 4%; 4 ± 1% and 3 ± 2%, respectively). There were significant contractions in *Muribaculum* (0.1 ± 0.0%; *P*<0.05) and 14 genera from the *Firmicutes* phylum, including but not limited to *Lactococcus* (1.8 ± 0.2%; *P*<0.001), *Acetatifactor* (2 ± 0.3%; *P*<0.001), *Kineothrix* (2 ± 0.4%; *P*<0.01), and *Oscillibacter* (1 ± 0.2%; *P*<0.001), in vehicle-treated mice compared with FCM- and FDI-treated mice (4 ± 1% and 5 ± 1%; 7 ± 1% and 9 ± 1%; 5 ± 1% and 6 ± 1%; 4 ± 1 and 5 ± 1%, respectively). In contrast, *Enterococcus* (0.1 ± 0.02% vs. 0.01 ± 0.00%; *P*<0.001) and *Erysipelatoclostridium* (2 ± 0.1% vs. 1 ± 0.2%; *P*<0.01) were significantly increased in vehicle-treated mice compared with FCM-treated mice. There were significant expansions in 5 genera, including but not limited to, *Alistipes* (5 ± 2%) and *Desulfovibrio* (1 ± 0.4%) in FCM-treated mice but not vehicle- or FDI-treated mice (0.02 ± 0.02% and 0.01 ± 0.00%; 0.00 ± 0.00% and 0.00 ± 0.00%, respectively; *P*<0.05 vs. FCM). *Eubacterium*, *Emergencia*, and *Anaerotruncus* were significantly expanded in FDI-treated mice compared with FCM-treated mice (1 ± 0.3% vs. 2 ± 0.4%, *P*<0.05; 0.06 ± 0.02% vs. 0.12 ± 0.03%, *P*<0.05; 0.3 ± 0.1% vs. 1 ± 0.2%, *P*<0.01, respectively).

Further microbiome analysis at the species level (Figure 4F and Table 1), after removing all unassigned species, showed that 449 species were detected and 262 (42%) were significantly different between vehicle and FCM and FDI treatments. Significant differences between FCM and FDI were observed in 33 species (approximately 8%). When removing species with the lowest occurrence (<0.01% abundance), 54 species were present in vehicle-treated mice, 95 species were present in FCM-treated mice, and 92 species were present in FDI-treated mice, which is consistent with the increased diversity in the FCM and FDI treatment groups.

The species *Akkermansia muciniphila* (31 ± 2% vs. 14 ± 2% vs. 12 ± 3%; *P*<0.001 vs. vehicle) and *Paramuribaculum intestinale* (10 ± 1% vs. 4 ± 1% vs. 3 ± 2%, *P*<0.01 vs. vehicle) were the most abundant in vehicle-treated mice and were significantly expanded compared with FCM- and FDI-treated mice. In contrast, there were 24 species that were significantly contracted in vehicle-treated mice compared with FCM- and FDI-treated mice, including but not limited to: *Lactococcus lactis* (2 ± 0.2% vs. 4 ± 0.5% vs. 5 ± 0.6%; *P*<0.005), *Acetatifactor MGBC129547* (1 ± 0.1% vs. 2 ± 0.4% vs. 3 ± 0.3%; *P*<0.05), *Kineothrix sp000403275* (2 ± 0.3% vs. 5 ± 0.9% vs. 5 ± 0.7%; *P*<0.01), *Oscillibacter* MGBC161747 (0.02 ± 0.01% vs. 2 ± 0.6% vs. 2 ± 0.5%; *P*<0.001), and *Romboutsia ilealis* (0.6 ± 0.1% vs. 1.1 ± 0.2% vs. 1.7 ± 0.2%; *P*<0.01). There were 8 species that were significantly expanded in FCM-treated mice compared with FDI-treated mice, including, but not limited to, *Duncaniella sp001689425* (0.4 ± 0.1% vs. 0.0 ± 0.0%; *P*<0.0001), *Alistipes sp002362235* (1.4 ± 0.6% vs. 0.0 ± 0.0%; *P*<0.05), and *Desulfovibrio MGBC000161* (0.8 ± 0.4% vs. 0.0 ± 0.0%; *P*<0.05). In contrast, five species were observed to be significantly contracted in FCM- compared with FDI-treated mice, including, but not limited to, *Eubacterium MGBC101131* (0.7 ± 0.3% vs. 1.8 ± 0.3%; *P*<0.05), *Dorea MGBC109699* (0.06 ± 0.01% vs. 0.15 ± 0.02%; *P*<0.0001), and *Oscillibacter sp000403435* (0.1 ± 0.01% vs. 0.4 ± 0.1%; *P*<0.05).

**Figure 5:**
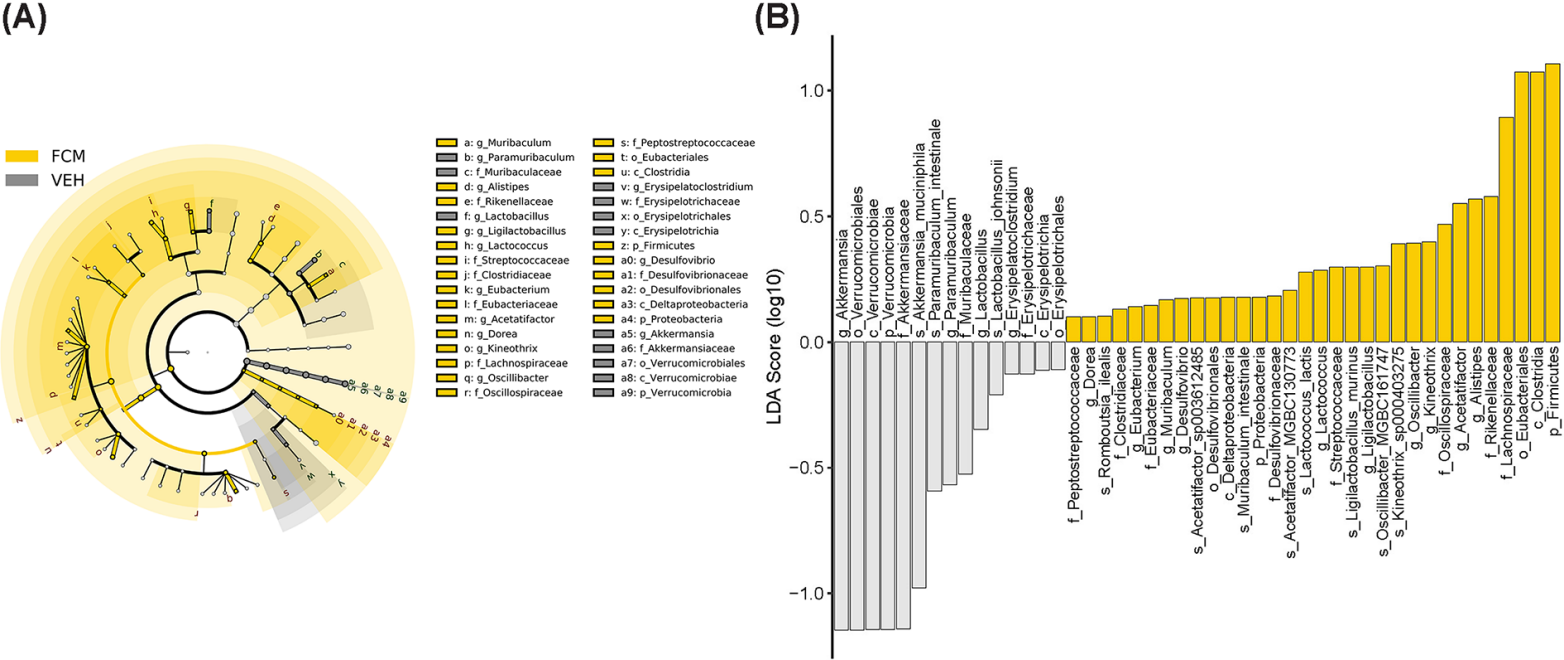
LEfSe analysis of gut microbiota from vehicle- and FCM-treated mice Cladograms (**A**) show the microbial clades with the greatest differences in the abundance of microbiota between vehicle- and FCM-treated mice. LDA scores (**B**) of microbial clades differing in abundance between vehicle- and FCM-treated mice (LDA score >0.1 and significance of *P*<0.05, determined using Kruskal–Wallis test); *N*=8–9/genotype.

**Figure 6:**
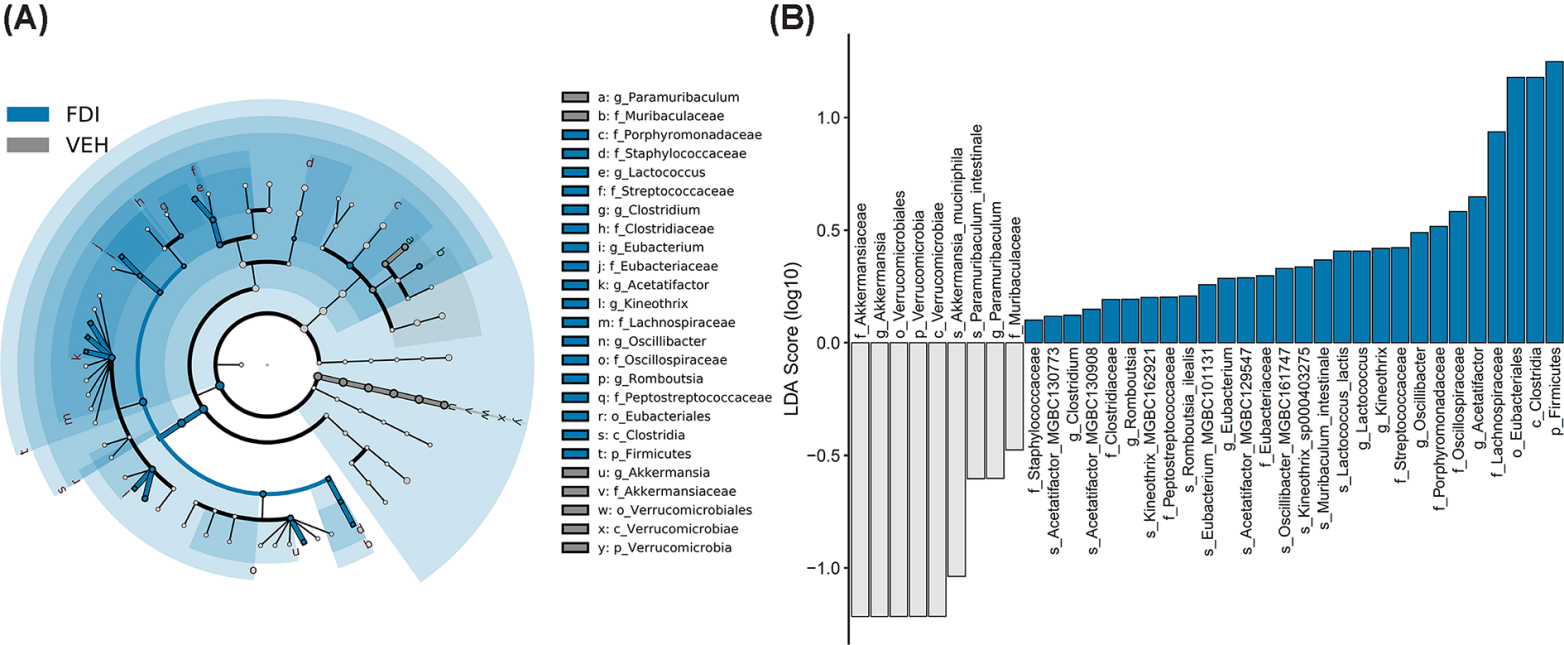
LEfSe analysis of gut microbiota from vehicle- and FDI-treated mice Cladograms (**A**) show the microbial clades with the greatest differences in the abundance of microbiota between vehicle- and FDI-treated mice. LDA scores (**B**) of microbial clades differing in abundance between vehicle- and FDI-treated mice (LDA score >0.1 and significance of *P*<0.05, determined using the Kruskal–Wallis test); *N*=8–9/genotype.

**Figure 7:**
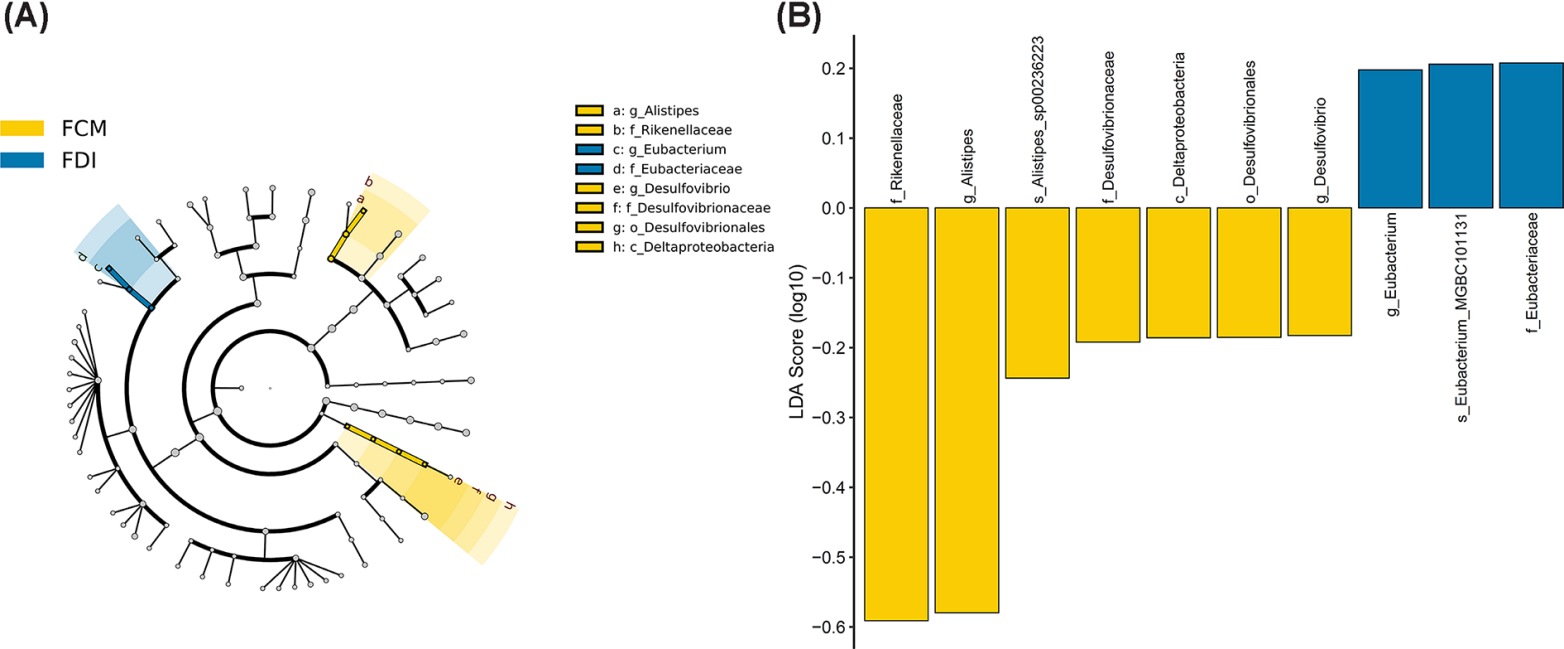
LEfSe analysis of gut microbiota from FCM- and FDI-treated mice Cladograms (**A**) show the microbial clades with the greatest differences in abundance in the microbiota from FCM- and FDI-treated mice. LDA scores (**B**) of microbial clades differing in abundance between FCM- and FDI-treated mice (LDA score >0.1 and significance of *P*<0.05, determined using the Kruskal–Wallis test); *N*=8–9/genotype.

Linear discriminant analysis effect size (LEfSe) identified the microbiota with the greatest differences in abundance between vehicle-treated mice and FCM- and FDI-treated mice (Figures 5 and 6). The phylum *Verrucomicrobia*, class *Erysipelotrichia*, genera *Paramuribaculum* and *Lactobacillus*, and family *Muribaculaceae* were significantly contracted in vehicle-treated mice compared with FCM-treated mice. Compared with FDI treatment, vehicle-treated mice also showed contractions in the phylum *Verrucomicrobia* and family *Muribaculaceae*. FCM treatment caused expansion of the phylum *Firmicutes*, which includes the genera *Muribaculum*, *Alistipes*, *Ligilactobacillus*, *Lactococcus*, *Eubacterium*, *Acetatifactor*, *Dorea*, *Kineothrix*, *Oscillibacter* and *Desulfovibrio*. Treatment with FDI caused expansion of the phylum *Firmicutes*, which included the genera *Lactococcus*, *Clostridium*, *Eubacterium*, *Acetatifactor*, *Kineothrix*, *Oscillibacter*, and *Romboutsia*. Specific differences were observed when comparing FCM with FDI treatment, wherein FCM induced an expansion in the family *Eubacteriaceae* and contractions in *Desulfovibrionaceae* and *Rikenellaceae* (Figure 7).

## Discussion

Emerging evidence suggests that the intestinal ionic milieu, including iron, can significantly affect the composition of the gut microbiome. Similarly, the state of the gut microbiome can influence iron homeostasis in the host. Several studies have shown that oral iron supplementation and dietary iron deficiency can alter the intestinal microbiota; however, studies focusing on the effect of IV iron supplementation on the gut microbiome in IDA have not been conducted. The goal of the current study was to determine how two IV iron therapy preparations, FCM and FDI, affect the gut microbiome in female mice with IDA. Using metagenomic shotgun sequencing to investigate with high resolution how the gut microbiome changes from phylum to species level, we were able to show that there is significant contraction and decreased microbial diversity in IDA and that IV iron replenishment leads to a bacterial ‘bloom’ and increased microbial diversity. Our data demonstrate that, in addition to dietary iron availability, IV iron administration can also influence the composition of the gut microbiome.

In mice, the gut microbiome is primarily composed of *Firmicutes* and *Bacteroidetes* [19]. However, in our vehicle-treated mice with IDA, the predominant phylum was *Verrucomicrobia* (∼47%), whereas *Firmicutes* and *Bacteroidetes* only comprised ∼53% of the microbiota. In contrast, replenishing iron stores in IDA with IV FCM or FDI restored the gut microbiome, such that *Firmicutes* and *Bacteroidetes* were the dominant phyla, comprising 78% and 83% of the microbiota in FCM- and FDI-treated mice, respectively. Interestingly, the abundance of *Bacteroidetes* was not different among the three treatment groups; however, *Firmicutes* abundance more than doubled, whereas *Verrucomicrobia* abundance was reduced by more than 50% when IDA mice were treated with either FCM or FDI. The *Firmicutes/Bacteroidetes* (F/B) ratio is used as an indicator of dysbiosis [20]; an increased F/B ratio has been observed in obesity [21], while a decreased F/B ratio is associated with the progression of intestinal diseases like IBD [22]. We found that vehicle-treated mice with IDA have a ∼50% lower F/B ratio compared with mice treated with either FCM or FDI. Of note, IDA is common in patients with IBD, and IV iron is recommended as the first choice of treatment in the case of active IBD [23]. A study comparing the effects of oral versus IV iron replacement therapy in patients with IBD found that despite similar clinical outcomes, there were clear oral- and IV-specific fingerprints in bacterial phylotypes and metabolome [24]. Consequently, any iron replacement therapy in these patients will have an impact on the composition of the gut microbiome. This could become particularly important in IBD patients receiving multiple or recurrent iron infusions and could have secondary consequences, via shifts in the gut microbiome, on disease activity.

One of the most striking findings in our study was that *Verrucomicrobia* accounted for nearly 50% of the relative abundance of phyla in vehicle-treated iron-deficient mice compared with IDA mice treated with FCM (∼20% relative abundance) or FDI (16% relative abundance). The expansion in *Verrucomicrobia* was mainly due to increased abundance of the species *Akkermansia muciniphila*, a mucin-degrading bacterium with probiotic propertie [25]. *A. muciniphila* is generally considered a ‘health promoting’ organism and is more abundant in the gut of healthy individuals than in patients with diabetes mellitus, obesity, intestinal diseases, and metabolic disorders [25]. Supplementation with *A. muciniphila* was found to reverse Western diet-induced exacerbation of atherosclerotic lesions in apolipoprotein E-deficient mice [26]. and reversed high-fat diet-induced metabolic disorders in obese and diabetic mice [27]. Until now, the role of iron in influencing abundance of *A. muciniphila* has never been studied. However, in 4-day fasted Syrian hamsters [28], and Burmese pythons subjected to food withholding for 30 days [29], *A. muciniphila* abundance significantly increased (both conditions associated with lack of dietary iron intake). Mucin-degrading bacteria have a competitive advantage during nutrient deprivation because they can utilize mucin as a constant source of nutrients. However, mucus production/secretion by the host was shown to correlate with dietary iron content, suggesting that secreted mucus can protect the host from excess iron absorption [30]. In our study, all mice, regardless of treatment group, were on an iron-deficient diet, which may have given *A. muciniphila* an advantage for growth in the vehicle-treated IDA mice. Interestingly, biliary iron excretion and enterohepatic recycling of non-transferrin-bound iron have been described in models of iron overload or when transferrin is saturated [31]. Therefore, we hypothesize that as plasma iron levels increased in the IV iron treatment groups, there was an increase in biliary iron excretion, providing a luminal source of required nutrients to other gut microbes. Consequently, the abundance of *A. muciniphila* was reduced in the FCM- and FDI-treated mice.

We observed significant contraction of *Firmicutes* in vehicle-treated mice with IDA, including contraction at the class (e.g., *Clostridia*), order (e.g., *Bacillales* and *Eubacteriales*), family (e.g., *Streptococcaceae, Clostridiaceae, Eubacteriaceae, Lachnospiraceae*, and *Oscillospiraceae*), genus (e.g., *Eubacterium, Acetatifactor, Kineothrix, Oscillibacter*, and *Romboutsia*), and species (e.g., *Eubacterium* spp., *Acetatifactor* spp., *Kineothrix* spp., *Oscillibacter* spp.) levels, compared with FCM- and FDI-treated mice. Of note, the genus *Lactobacillus* is important for determining host iron absorption because the lactic acid that is produced by *Lactobacilli* affects dietary iron bioavailability [32]. *Lactobacilli* sense luminal iron levels and, via a complex mechanism involving inhibition of hypoxia-inducible factor 2α by microbial metabolites, can attenuate iron absorption [33]. Depending on the study, *Lactobacillus* was found to increase, decrease, or remain stable in response to different dietary iron content [34–38]. Surprisingly, *Lactobacilli* themselves do not require iron for growth [39,40]; consistent with this, our study did not show significant differences in abundance of *Lactobacillus* between vehicle-, FCM- or FDI-treated groups. In addition, rat-fed diets with different iron content also did not show significant differences in *Lactobacillus* abundance [13]. Of note, these data and our data contrast with studies in iron-deprived mice and young Sprague Dawley rats, which showed that *Lactobacillus* abundance was significantly increased compared with animals on iron-supplemented diets [13,34]. The latter might indicate differences depending on the age of the animal. In contrast, in Indian women with IDA, the species *Lactobacillus acidophilus* was significantly reduced compared with women with normal hemoglobin levels; however, diet was not sufficiently controlled in this study [41]. Whether these observations are direct effects of iron or if iron-induced shifts to the intestinal microenvironment can lead to selective pressure on microbiota, ultimately leading to certain microbes gaining a growth advantage, while others become restricted, remains to be determined.

A few important differences were observed between the FCM and FDI treatments, with approximately 8% of the species showing significance. Interestingly, these differences occurred despite a similar correction of Hct and RBC between the FCM and FDI treatment groups. The abundance of the phylum *Proteobacteria* was ∼20-fold higher in FCM-treated mice than in vehicle- or FDI-treated mice, mostly due to a significant expansion in the species *Desulfovibrio MGBC000161*, which was only detectable in mice treated with FCM. Of note, *Desulfovibrio* spp. are sulfate-reducing bacteria that have been shown to directly reduce ferric iron [42]. Further, *Desulfovibrio* was shown to increase in abundance in the cecum of piglets fed a high-iron diet [43]. Similarly, several members of the phylum *Bacteroidetes* were also found to be more abundant in FCM-treated mice than in FDI-treated mice, including *Duncaniella sp001689425, Phocaeicola vulgatus*, and *Alistipes sp002362235* (recently published under the name *Alistipes okayasuensis*) [44]. Interestingly, the abundance of the genus *Alistipes* was more than 500-fold higher in FCM-treated mice than in vehicle- and FDI-treated mice. *Alistipes finegoldii* was shown to be more abundant when mice were fed an iron-supplemented diet after antibiotic exposure [45]. *Alistipes* was also shown to be decreased in growing rats fed a low-iron diet compared with a control diet [46]. In contrast, several *Clostridia* members from the phylum *Firmicutes* were found to be expanded in FDI-treated mice compared with FCM-treated mice. These included *Dorea MGBC109699 and Dorea MGBC000089, Eubacterium MGBC101131, Oscillibacter sp000403435*, and *Emergencia MGBC000042*. The genus *Eubacterium* is frequently encountered in the intestinal tract of humans and mice [47]. We found that FDI-treated mice had ∼2-fold higher abundance in *Eubacterium* compared with mice treated with FCM. Some *Eubacterium* spp. have been described as key producers of short-chain fatty acids and play important anti-inflammatory roles by inhibiting pro-inflammatory cytokines. In patients with IBD, *Eubacterium* spp. are consistently reduced [48,49], and these patients also show a less diverse microbiome compared with healthy individuals; however [50], we do not yet know what accounts for the differences observed between the two IV iron preparations. Importantly, we did not observe differences in plasma iron levels between FCM and FDI treatment groups, suggesting that the differences in the microbiome between these two groups cannot be attributed to varying plasma iron levels. However, we hypothesize that it may be related to the influence of the different carbohydrate moieties that help stabilize the iron core on the gut microbiota.

It is known that iron availability in the intestinal lumen can promote the replication and virulence of enteric pathogens such as *Salmonella, Shigella*, and *Campylobacter* [51,52]. In contrast, luminal iron availability has also been shown to attenuate the virulence of some enteric pathogens such as *Citrobacter* [53]. However, we did not detect enteric pathogens in our study of IDA. For example, *S. typhimurium* was completely absent and *C. difficile* was far below 0.01% abundance in all treatment groups.

Our study has some limitations. First, we evaluated the effects of IV iron supplementation on fecal microbiota; however, due to the nature of how iron is transported in the intestine and the general segment-specific composition of the gut microbiome, it remains to be determined whether there are differences in the microbial signatures between the small and large intestines and between mucosa-associated and fecal microbiota. Therefore, further studies are required. Second, owing to the high prevalence of IDA in women, we only utilized female mice in our study. Consequently, we could not draw any conclusions regarding sex differences. Third, the composition of the gut microbiome can be significantly different depending on the animal facility where the mice are housed [54]; thus, we cannot exclude facility-specific effects in our mouse colony. Despite these limitations, our data provide novel insights into changes in the gut microbiome in response to the correction of IDA with IV iron. Furthermore, we were able to show that even though both IV iron preparations equally corrected iron deficiency, there were subtle (∼8%) but significant differences in how FCM vs. FDI affected the gut microbiome. More detailed studies and longer observational periods are required to better understand if these two IV iron preparations affect microbiome composition in humans. However, the present study demonstrated that IV iron supplementation affects the gut microbiome and consequently contributes to altered disease outcomes, particularly in clinical conditions such as IBD and chronic kidney disease.

## Data Availability

The dataset supporting the conclusions of this article is available at https://doi.org/10.6084/m9.figshare.23523639.

## Abbreviations

DSS: dextran sulfate sodium
FCM: ferric carboxymaltose
FDI: ferric derisomaltose
IBD: inflammatory bowel disease
IDA: iron-deficient anemia
LDA: linear discriminant analysis
LEfSe: linear discriminant analysis effect size
NGS: next-generation sequencing
NS: not significant
OTU: operational taxonomic units
PCoA: principal coordinates analysis
PERMANOVA: permutational multivariate analysis of variance
